# Evaluation of the quality of life in patients with substance use disorders – A suggested algorithm

**DOI:** 10.1192/j.eurpsy.2025.1002

**Published:** 2025-08-26

**Authors:** V. A. Voicu, O. Vasiliu

**Affiliations:** 1 Carol Davila University of Medicine and Pharmacy; 2 Romanian Academy of Medical Sciences; 3 Romanian Academy; 4 Psychiatry Dept., Dr. Carol Davila University Emergency Central Military Hospital, Bucharest, Romania

## Abstract

**Introduction:**

Assessment of the quality of life (QoL) in patients diagnosed with substance use disorders (SUDs) is important due to the need to explore potential ways of improving the prognosis and evolution of these patients towards recovery and prolonged abstinence. The impact of SUDs on the QoL is often difficult to determine in the absence of structured methods because of the multiple factors that may moderate the results, including misperceptions of the patients on the effects of the drugs of abuse (DOA), stigmatization, countertransference aspects of the therapeutic relationships, etc.

**Objectives:**

To assess the most validated psychometric tools dedicated to the evaluation of QoL in patients with SUDs.

**Methods:**

A literature review was conducted in three electronic databases (PubMed, Google Scholar, and Clarivate/Web of Science) to find clinical reports published between January 2000 and September 2024 regarding the QoL instruments in patients with SUD. The keywords used were “substance use disorders”, “quality of life”, “health-related quality of life”, “scales”, “inventories”, and “questionnaires”. There was no restriction regarding the age of participants, and only sources published in English were selected.

**Results:**

The most frequently reported scales used for the purpose of monitoring the QoL in patients with SUDs were the Short-Form Health Survey (SF-36) and EuroQoL (EQ) 5D 5L. Also, WHOQOL-BREF, which is associated with the WHO International Classification of Diseases system, is a comprehensive and useful instrument that may be applied in patients with SUDs. Health-related Quality of Life for Drug Abusers (HRQOLDA) and Injection Drug Use Quality of Life Scale (IDUQOL) may be used in this specific population because they refer more exactly to daily life aspects of the patients with SUD that interfere with their QoL, but these instruments are less known and applied by clinicians.

**Conclusions:**

General instruments, like surveys or inventories, targeting the evaluation of QoL and, also, more specific instruments focused on the quality of the daily life of patients with SUDs need to be administered in case management because these may highlight important areas of therapeutic intervention. The inclusion of such instruments is expected to have multiple benefits, such as better treatment adherence through the improvement of the therapeutic relationship, more targeted interventions for the meeds of this population, etc.

**Disclosure of Interest:**

None Declared

